# Management of hidradenitis suppurativa in UK primary care: a cross-sectional survey

**DOI:** 10.3399/BJGPO.2025.0060

**Published:** 2025-12-19

**Authors:** Hannah E Wainman, Stephanie Gallard, Matthew J Ridd, John R Ingram

**Affiliations:** 1 Bristol Dermatology Centre, Bristol Royal Infirmary, University Hospitals Bristol and Weston NHS Foundation Trust, Bristol, UK; 2 Centre for Applied Excellence in Skin & Allergy Research, University of Bristol, Bristol, UK; 3 Royal Liverpool University Hospital, Liverpool University Hospitals NHS Foundation Trust, Liverpool, UK; 4 Division of Infection & Immunity, Cardiff University, Cardiff, UK

**Keywords:** hidradenitis suppurativa, primary health care, dermatology, qualitative research

## Abstract

**Background:**

Hidradenitis suppurativa (HS) is a painful, chronic, inflammatory skin condition affecting the skin folds. It is frequently misdiagnosed, leading to delays in care and the progression of the disease to permanent scarring.

**Aim:**

To understand the level of knowledge and confidence of healthcare professionals (HCPs) in primary care managing patients with HS. To establish their ability to recognise the early signs of HS, awareness of associated comorbidities, and recognition of treatment options available in primary care.

**Design & setting:**

A survey was distributed to HCPs working in primary care in the UK.

**Method:**

The survey was disseminated via weekly GP bulletins distributed by local integrated care boards, the Primary Care Dermatology Society (PCDS) mailing lists, and at professional events.

**Results:**

Of 183 responders, most (93%) did not have a specialist role in dermatology or a postgraduate qualification in dermatology (69%), 36 (20%) were not doctors, and there was a good geographical spread over the UK. Of the responders, 74% felt confident diagnosing HS, but only 39% were confident in managing the pain associated with the disease. Perceived confidence did not correlate with understanding the importance of early referral to secondary care where multiple skin sites were affected.

**Conclusion:**

Further education in diagnosing and managing HS in primary care is needed. Future research could focus on developing a tool to support the diagnosis of HS in primary care and a clear, primary care-focused management guideline for identified patients.

## How this fits in

Hidradenitis suppurativa (HS) has a diagnostic delay of 7–10 years, and healthcare professionals (HCPs) in primary care are ideally placed to make the diagnosis, start initial therapy, and support the management of associated comorbidities. Limited work has been done to understand the diagnosis and management of HS in primary care and focused on only GPs and those with a specialist interest in dermatology. This survey showed that 74% of HCPs in primary care are confident in making an initial diagnosis but are less confident in starting therapy and managing associated pain. The results confirm the need for collaborative guidelines and learning material to support managing patients with HS.

## Introduction

**Figure 1.**
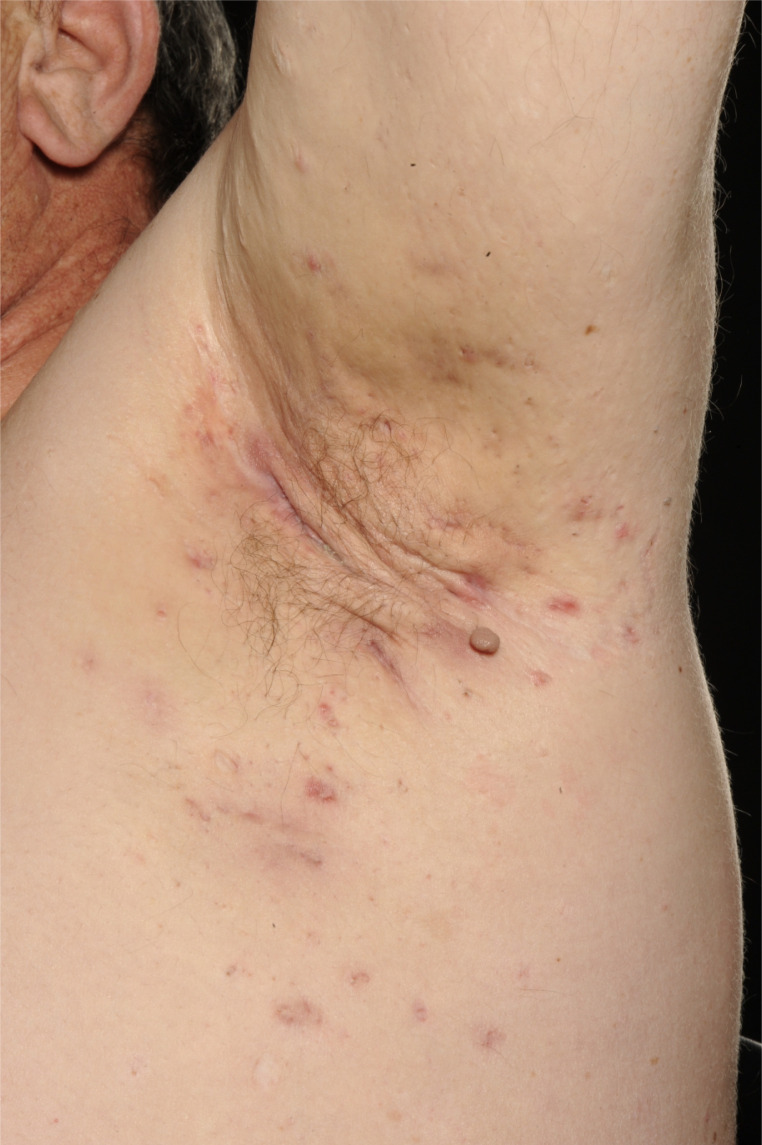
Typical presentation of HS showing rope-like scarring, double-ended comedones, tombstone scarring and nodules

Hidradenitis suppurativa (HS) is a chronic, painful, inflammatory skin condition that begins with nodules and abscesses in the skin folds and progresses to tunnels and scarring (Figure 1). It affects 1%–4% of the population and has an average delay to diagnosis of 7–10 years.^1–4^

Patients report a lack of knowledge of HS by healthcare professionals (HCPs) and struggle to get referred to secondary care as the disease progresses.^5^ Patients often feel misunderstood, unheard, and dismissed.^6^ HCPs frequently advise lifestyle changes, such as smoking cessation, weight loss, or hygiene practices, without understanding the challenges for the individual, resulting in feelings of shame and disengagement from health services.^6^ HS is associated with many comorbidities, including cardiovascular disease, depression, and diabetes. In addition to initiating treatment and referral early, HCPs in primary care are best placed to diagnose and manage these comorbidities.

The average patient will see five clinicians and receive three incorrect diagnoses before being diagnosed with HS.^1,3^ Most patients have moderate-to-severe disease at the time of diagnosis. An Australian survey suggested that 10% of patients self-diagnose their HS.^7^ The most common incorrect diagnoses include folliculitis and furuncles (boils and cysts).^8,9^

HCPs working in primary care need an improved ability to recognise the clinical signs of the disease to facilitate, through timely referral, prompt review of patients with HS in secondary care. This ensures access to more definitive therapy, such as biologics, before the disease progresses to scarring. Evidence supports a ‘window of opportunity’ for starting biologic therapy.^10^ Given the current long waiting times for review in secondary care, a heightened awareness of treatment options available in primary care is imperative to improving patient outcomes.

This study aimed to understand the level of actual and perceived knowledge of HCPs in primary care who manage patients with HS. It aimed to establish their recognition of the early signs of HS, understanding of the associated comorbidities, and recognition of the treatment options available in primary care.

## Method

### Development of survey

The survey was developed with the support of the Primary Care Dermatology Society (PCDS) committee and piloted on a group of GPs who were not involved in the development process.

The survey used the Jisc Online Surveys platform (www.jisc.ac.uk), which allowed distribution using a Quick Response (QR) code or Uniform Resource Locator (URL). Results were collected anonymously, but participants were given the opportunity to share their email addresses at the end of the survey to be involved in future research in this area.

### Distribution and sampling

An important aspect of this work was ensuring that HCPs in primary care without a specific interest in dermatology were sampled. The survey was distributed widely using regional weekly GP bulletins distributed by local integrated care boards in Gloucestershire, Bristol, North Somerset, South Gloucestershire, Liverpool, and Merseyside, where the authors had affiliations to support distribution. It was shared at dermatology teaching events, including the Southwest Skin Club and the PCDS mailing list.

### Data collection

The survey was open from 29 February 2024–29 June 2024. A response rate cannot be calculated as the survey was distributed widely to promote uptake and allow for convenience sampling.

### Survey design

The survey had the following three parts:

1. Demographic information about the participants' and responders’ experience in dermatology.
2. Responders’ perceived knowledge of HS, particularly their confidence in diagnosing, starting therapy, recognising comorbidities, and managing pain.
3. Knowledge was tested with clinical scenarios and questions.

The survey used a mix of free-text answers and multiple-choice options. To encourage completion, not all questions were mandatory. A full list of the questions asked is shown in Supplementary Appendix 1.

### Data analysis

We aimed to recruit at least 100 participants to obtain a range of views and participant characteristics. Data were interpreted using the JISC analysis platform and Excel; qualitative data were analysed using NVivo 15 software.

## Results

### Demographics and dermatology experience

Of the 183 responders, 178 (97%) completed all questions. The demographics of the responders are shown in Table 1. Most responders (93%) did not have a specialist role in dermatology or a postgraduate qualification in dermatology (69%), 36 (20%) were not doctors, and there was a good geographical spread across the UK. Response percentages are calculated according to the number of responses and not the overall sample size.

**Table 1.** Demographics of responders, *N* =183

Demographic		*n* (%)
**Clinical role**	GP	129 (70)
	Practice nurse	1 (0.5)
	Advanced nurse practitioner	19 (10)
	Physician associate	2 (1)
	Pharmacist	1 (0.5)
	District nurse	1 (0.5)
	Specialist nurse in dermatology (community)	8 (4)
	GP with extended role in dermatology	7 (4)
	Paramedic	3 (2)
	GP resident	12 (7)
**Length of time in current role**	<1 year	15 (8)
	1–2 years	22 (12)
	3–5 years	30 (16)
	5–10 years	30 (16)
	>10 years	86 (47)
**Geographical area**	North East	26 (14)
	North West	14 (8)
	Yorkshire and Humber	12 (7)
	East Midlands	4 (2)
	West Midlands	7 (4)
	East of England	14 (8)
	London	19 (10)
	South East	24 (13)
	South West	54 (30)
	Scotland	5 (3)
	Wales	4 (2)
**Postgraduate qualification in dermatology**	Yes	57 (31)

### Perceived confidence in managing HS

Of the responders, 21% regularly saw patients with HS in their practice, 54% occasionally, and only 3% never saw patients with HS. In the last month, 71% saw one or fewer patients with HS compared with 4% seeing four or more patients.

Overall, 74% of responders reported feeling confident making an initial diagnosis of HS, with 67% feeling happy to start initial treatment. Only 39% felt confident managing pain in HS, and 45% felt confident identifying and managing the comorbidities associated with the condition. Confidence results divided into GPs and other HCPs are shown in Table 2.

**Table 2.** Percentage confidence of responders in managing aspect of hidradenitis suppurativa

Perceived confidence in	GPs excluding residents, %	Other HCPs, %
Diagnosing HS	81	57
Starting initial therapy	70	61
Managing pain	40	35
Identifying and managing comorbidities	50	35

HCPs = healthcare professionals

### Assessed knowledge

Responders were given the following clinical scenario:

A 25-year-old patient presents to you with an abscess in her right axilla; she describes it as painful and has been there for 1–2 months. Her notes don't report any previous presentations with abscesses. How would you manage her? (Pick the single best answer.)

Of the 182 responders, 86% managed this patient with a course of antibiotics and a repeat review, 7% offered an incision and drainage, 1% referred the patient to the emergency department, and 7% diagnosed the patient with HS overall. A single episode of an abscess without further information does not confirm a diagnosis of HS as recurrent episodes define HS.

Of those who self-reported being confident in managing HS, 15% opted to book for an incision and drainage, refer to the emergency department, or diagnose HS instead of a course of antibiotics and a repeat review, which would be the most appropriate management in this scenario.

Of those reporting confidence in diagnosing HS, 15% didn’t feel confident in starting initial therapy. Only 46% of those confident in managing HS referred to secondary care when a patient had multiple sites affected.

Free-text answers to the question ‘*What clinical signs do you look for to point to a diagnosis of HS?*’ were analysed to identify common words, using simple word counts. Table 3 shows the top 10 words.

**Table 3.** Common words used to identify the clinical presentation of hidradenitis suppurativa, *N* = 159

Word	*n*
**Abscess**	68
**Scarring**	59
**Groin**	50
**Recurrent**	39
**Multiple**	36
**Axilla**	36
**Nodules**	25
**Boils**	19
**Comedones**	18
**Sinus**	17

**Figure 2.**
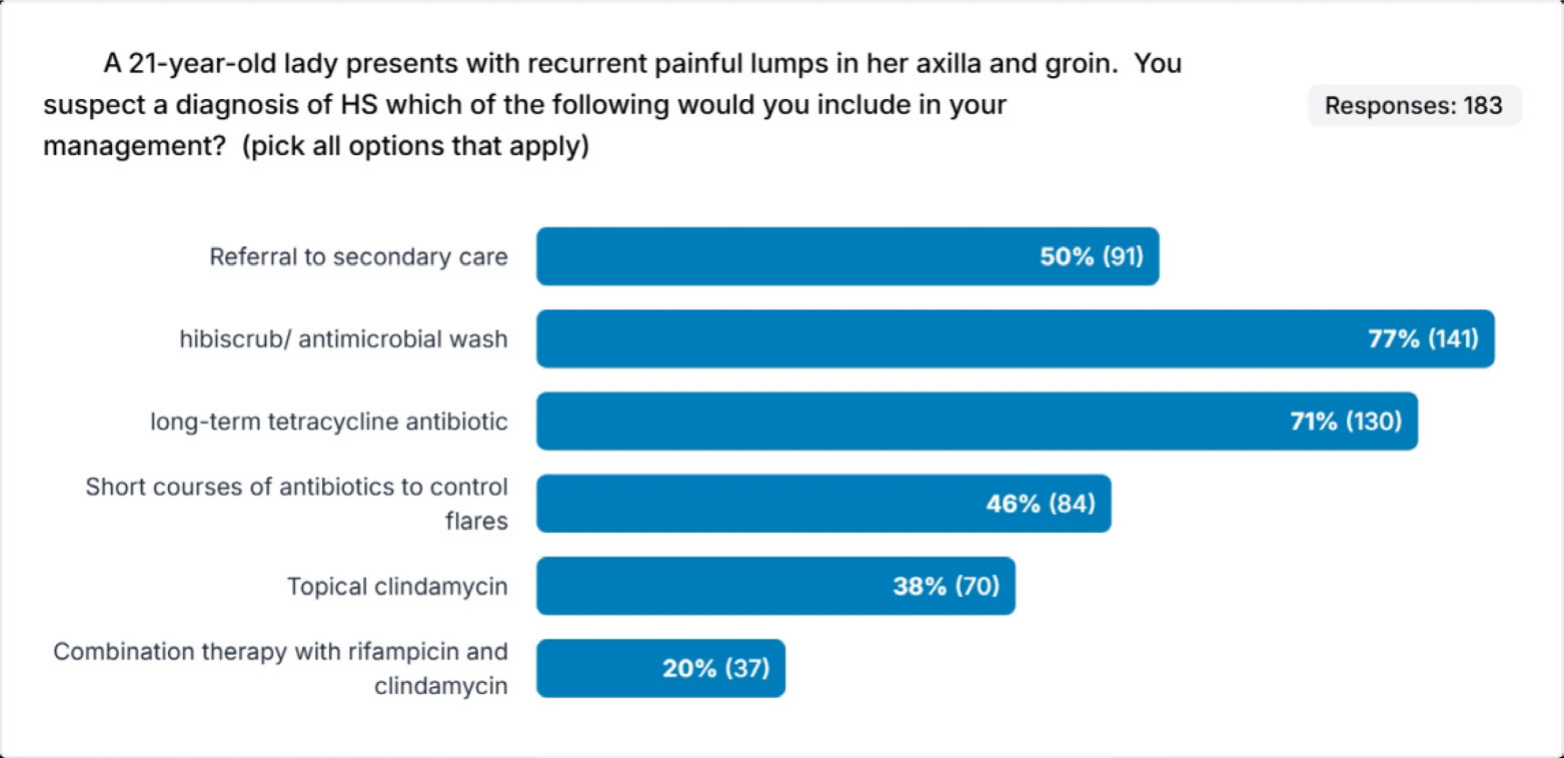
Bar chart showing responses to the management of a patient with HS at multiple sites who has not been diagnosed with HS before. HS = hidradenitis suppurativa. Note: Hibiscrub discontinued and replaced with Hibiwash.

Figure 2 shows the responses when given options for a patient’s first-line treatment in a new diagnosis of HS occurring at multiple sites.

Managing the pain associated with HS showed that 63% would prescribe non-steroidal anti-inflammatory medication, 24% neuropathic painkillers, and 4% opiates. There is currently no consensus on the best way to manage pain in HS.

To assess the number of dressings routinely prescribed, the responders were given a scenario where a patient had discharging nodules in both axillae and asked how many dressings they would provide for a month. Thirty per cent would provide 30 or fewer dressings, leaving the patient with less than one dressing per axilla per day. Overall, GPs prescribed smaller amounts of dressings than non-GPs, with 78% of non-GPs prescribing more than 60 dressings per month compared with 67% of GPs.

Responders were given a pick list with some distractors (menorrhagia, lichen planus, and renal failure) to assess their knowledge of comorbidities associated with HS. Table 4 shows the results for the identification of common comorbidities. None of the responders correctly identified all of the comorbidities. However, the list of included comorbidities was not meant to be exhaustive.

**Table 4.** Identification of the comorbidities by responders, *N* = 178

Comorbidity	Identified, *n* (%)
**Cardiovascular disease**	126 (71)
**Non-alcoholic fatty acid disease**	102 (57)
**Down syndrome**	25 (14)
**Thyroid disease**	57 (32)
**Suicide**	91 (51)
**Anxiety**	124 (70)
**Depression**	140 (79)
**Alcoholism**	68 (38)
**Inflammatory bowel disease**	90 (51)
**Type 2 diabetes**	149 (84)
**Polycystic ovarian syndrome**	138 (78)
**Pyoderma gangrenosum**	25 (14)
**Pilonidal sinus**	84 (47)
**Acne**	94 (53)
**Anaemia**	18 (10)
**Psoriasis**	23 (13)
**Scalp folliculitis**	45 (25)

A question exploring understanding of the evidence to support treatment options in HS highlighted that 65% of responders felt that the greatest evidence-based treatment was weight loss or smoking cessation. Other options included antimicrobial washes, tetracyclines, and referral to secondary care for biologics or systemic therapy.

## Discussion

### Summary

The results show a lack of concordance between primary care HCPs' self-rated and objective knowledge of HS, highlighting an ongoing educational need. They showed that while many HCPs may recognise HS, they are unsure of the next steps in management and particularly lack confidence in pain management. The lack of confidence in pain management may not be specific to HS and may instead reflect a general lack of consensus on the management of chronic pain in general.

The results show that HCPs in primary care are well-informed about the more common comorbidities such as diabetes (84%) and depression (79%). They are less aware of associations such as pilonidal sinus (47%) and acne (53%). Patients presenting with the latter afford a good opportunity to screen for HS and could support earlier diagnosis.

### Strengths and limitations

This survey incorporates the views of the wider primary care healthcare team, beyond GPs, and attempts to assess knowledge rather than relying on perceived knowledge levels. Furthermore, in contrast to the previous UK-based GP survey,^11^ most did not have a specialist role in dermatology.

As with any widely distributed survey, which is not mandatory, responders may be more interested and/or knowledgeable with the topic than non-responders. This can lead to skewed responses that may overinflate the target group’s knowledge level. The low proportion of responders with dermatology postgraduate qualifications (31%) suggests that most responders did not have a specialist interest in dermatology. The survey was opportunistically shared at dermatology events and via the PCDS mailing list, meaning that even if a specific qualification did not exist, participants would likely be interested in dermatology. To mitigate for this, the survey was also deliberately shared through other mailing lists, but data on PCDS membership was not collected. Furthermore, as the survey was explicitly about HS, responders already have HS at the forefront of their minds when formulating diagnoses. A further study assessing GP knowledge of various dermatology conditions may give a better understanding of their abilities to diagnose HS.

Over the past 10 years, the workforce in primary care has diversified with fewer GPs per 1000 patients and increased utilisation of multidisciplinary teams.^12^ This study included the wider primary care team. Twenty per cent of responders were not doctors. Increasingly, primary care practices have multiple different practitioners assessing and managing patients with skin disease, and it is paramount to include their views and experiences for a holistic view of the workforce knowledge.

### Comparison with existing literature

Research in HS has increased exponentially over the past 10 years. It could be postulated that recognition would also improve with the increased focus on the disease. Unfortunately, the current study shows that this is not the case. Confidence in diagnosing HS among GPs alone was lower in this cohort than in the previous study in 2021.^11^ That confidence is even lower among allied HCPs and residents in primary care. This reflects the repercussions of a lack of dermatology training for HCPs in primary care and the low priority given to skin disease. Where teaching is delivered, it is often heavily focused on skin cancer identification.^13,14^ Most of HS research has been published in dermatology journals, reducing information dissemination to non-dermatologists.^15^

In a 2021 survey of GPs, 94% were confident in diagnosing HS, but 60% self-reported a specialist interest in dermatology. Responders were asked about treatment options but were not assessed on their actual knowledge using clinical scenarios.^11^ Data from routine primary care electronic medical records contradicts these results by showing that one-third of patients with likely HS (recurrent flexural skin boils) were not formally diagnosed.^16^

Compared with previous work carried out in Denmark, where an unambiguous case was used and 85% of responders were able to diagnose HS,^17^ we used questions about clinical signs and management options to better understand responders’ knowledge.

HS involves self-management, including dressings, weight loss, and pain management. This has significant cost implications for the patient. Active HS can produce copious discharge, requiring frequent dressing changes.^18^ Patients report not being prescribed enough dressings, so they must supplement by purchasing their own. One study reported that over 40% of patients could not afford the number and quality of dressings required for their HS.^19^ By prescribing too few dressings, patients compensate with ‘homemade’ dressings and purchase their own, resulting in financial implications.^20^ Patients who struggle to control odour and discharge are more likely to withdraw from social activities and work owing to feelings of embarrassment and shame.^6^

### Implications for research and practice

As both HCPs in secondary care and primary care lack the confidence to manage pain in HS,^21^ further work is needed to address pain management in HS.

If patients with HS are diagnosed with the condition earlier in their trajectory, it has been shown that they develop fewer comorbidities and treatment options are more effective. This has a knock-on effect on the health economy, as patients with HS have a high healthcare burden that increases with disease severity.^20^

Secondary and primary care must work together to support this complex patient group. With increasing government targets to move care from secondary to primary care, all HCPs need to advocate for our patients to ensure they receive timely intervention and ongoing support with managing comorbidities and prescribing. This can only be done effectively if we understand the expertise and knowledge of both groups. By identifying gaps in knowledge, we can develop tailored resources to support our primary care colleagues.

These results illustrate an ongoing need for further education in diagnosing and managing HS in primary care. HCPs in primary care have unprecedented pressures on their time, so any resources will need to be developed in conjunction with them to ensure they are concise and focused. As non-GPs form an increasing proportion of the primary care workforce, their engagement with any intervention would be essential.

Future research could focus on developing a tool to support the diagnosis of HS in primary care, with a clear, primary care-focused management guideline for those patients identified.

## Data

The dataset relied on in this article is available from the corresponding author on reasonable request.
